# Wernicke–Korsakoff syndrome associated with mtDNA disease

**DOI:** 10.1177/1756286420938972

**Published:** 2020-07-30

**Authors:** Idris Janos Jimoh, Barbara Sebe, Peter Balicza, Mariann Fedor, Ilona Pataky, Gabor Rudas, Aniko Gal, Gabriella Inczedy-Farkas, Gyorgy Nemeth, Maria Judit Molnar

**Affiliations:** Semmelweis University of Medicine, Budapest, Hungary; Gedeon Richter Nyrt, Budapest, Hungary; Semmelweis University of Medicine, Budapest, Hungary; Semmelweis University of Medicine, Budapest, Hungary; Peter Pazmany Catholic University, Budapest, Hungary; Semmelweis University of Medicine, Budapest, Hungary; Semmelweis University of Medicine, Budapest, Hungary; Northwell Health, NY, USA; Gedeon Richter Nyrt, Budapest, Hungary; Semmelweis University of Medicine, Üllői 26, Budapest, 1085, Hungary

**Keywords:** ataxia, cariprazine, encephalopathy, MELAS, memory impairment, mitochondrial disease, OXPHOS, schizophrenia, Wernicke Encephalopathy

## Abstract

**Introduction:**

Wernicke encephalopathy (WE) and Wernicke–Korsakoff syndrome (WKS) are well-known disorders caused by thiamine deficiency. In addition to the classical concept of these diseases, some literature data suggest a connection between mitochondrial dysfunction and WE/WKS. Psychotic disorders and WKS seem to run in families, as the deficiency of the oxidative phosphorylation can be a trigger factor in psychotic events and WE/WKS as well. We present a patient harbouring the m.A3243G mtDNA mutation with the clinical and magnetic resonance imaging (MRI) findings of WKS who developed schizophrenia with predominantly negative symptoms some years later.

**Case presentation:**

A 27-year-old woman was referred to our clinic with severe weight loss after severe vomiting episodes, memory dysfunction and gait ataxia. Family history, as well as clinical, imaging and laboratory findings suggested a mitochondrial aetiology of her symptoms. Brain MRI detected bilateral mild thalamic lesions and loss of corpus mammillae, indicating Wernicke encephalopathy. Genetic testing detected an m.A3243G mtDNA mutation, which has been frequently associated with mitochondrial encephalopathy with lactic acidosis and stroke-like episodes. High-dose vitamin B1 supplementation with supportive antioxidant therapy improved the patient’s memory and learning disturbance; however, some months later she developed psychosis with predominantly negative symptoms and her cognitive functions deteriorated again. Both cognitive and negative symptoms responded well to cariprazine monotherapy.

**Discussion:**

Mitochondrial disease due to mtDNA alteration can be a rare cause of WE. In addition to vitamin B1 supplementation, cariprazine with significant dopamine D3 receptor binding can be useful to treat the predominantly negative symptoms and cognitive dysfunction in patients with mitochondrial dysfunction.

**Conclusion:**

We assume that patients with a mitochondrial disorder might be prone to develop WE/WKS and therefore need tailored supportive therapy during metabolic crisis as well as symptom-based personalized antipsychotic treatment.

## Introduction

Originally, Wernicke described an encephalopathy with the clinical triad of ophtalmoparesis with nystagmus, ataxia and confusion. Soon after, Korsakoff described amnesia occurring in certain cases of chronic alcoholism. More than half a century elapsed before the common aetiology of thiamine deficiency was discovered as the cause of the signs, symptoms and link between the conditions of Wernicke’s encephalopathy (WE) and Korsakoff’s syndrome (KS). Wernicke– Korsakoff syndrome (WKS) has an acute phase of encephalopathy, which results in bilateral lesions of the periventricular nuclei, thalami and structures of Papez’s circuit, notably the mammillary bodies, and a chronic phase which resolves into relatively permanent bilateral lesions and endures global amnesia. In many cases, psychotic episodes were associated with the previously mentioned symptoms.^1^

WE and WKS are often underdiagnosed^2^ because even if the classical triad is present, physicians consider WE mostly in cases when the patient has a history of alcohol use disorder, which is indeed a common but not the only cause of thiamine deficiency. The importance of thiamine supplementation was elucidated by a study, which showed that the prevalence of WE decreased since the introduction of thiamine fortified flour in Australia.^3^ Non-alcohol-related WE is mostly associated with cancer, gastrointestinal (GI) surgery or disease, fasting, acquired immuno deficiency syndrome (AIDS) and malnourishment, which is underdiagnosed.^4^ In some cases, thiamine supplementation does not provide full recovery, and some patients develop WKS even if therapy is started in the acute phase.^5^ A putative genetic aetiology of WKS has been investigated. An alteration of the nuclear SLC19A3 gene (encoding thiamine transporter 2) was described to cause an increased risk of cerebral damage in dogs due to brain cells’ particular reliance on thiamine pyrophosphate (TPP)-dependent metabolism involved in energy metabolism and neurotransmitter synthesis. The observed severe TPP-dependent enzyme deficiency led to mitochondrial dysfunction, impaired oxidative phosphorylation (OXPHOS) and cellular injury. Some of the findings were similar to human WKS, however, several differences were also noted.^6^

A family with the mitochondrial 3243A gene (m3243A-G) mutation was reported as inherited thiamine deficiency and cardiac symptoms by Sato *et al.* Thiamine treatment decreased the serum concentrations of lactate and pyruvate and improved the cardiac symptoms. In this paper, nothing relating to WKS symptoms were reported.^7^

Mitochondrial dysfunction has also been reported in cases of mental disorders, such as schizophrenia.^8^ Positive and negative symptoms are associated with hyper- and hypodopaminergic activation of the mesolimbic and mesocortical pathways. Mitochondrial concentrations in these areas and mutations within the mtDNA are active areas of neuropsychiatric research. It could be that those areas are either particularly vulnerable to changes in oxidative stress or the risk of neuronal death is higher. The understanding of these mechanisms could aid new therapeutic approaches to the psychotic symptoms.^9,10^ There is an interesting link between mitochondrial dysfunction and primary Fahr disease with psychiatric symptoms, however, not all Fahr disease is associated with mitochondriopathies. In many of them the pathogenesis is still unclear.^11^ In the diagnostic workup of schizophrenia, several organic disorders have to be ruled out.

In this article, we describe the case of a young woman with a classical mitochondrial DNA disease (m.A3243G) presenting with signs of WKS to provide additional insight into the molecular and genetic mechanisms of WKS and the development of prominent negative symptoms in schizophrenia.

## Case report

The 32-year-old patient had normal childhood development. Her mother and maternal grandparents had diabetes mellitus, her mother suffered from exercise intolerance, hypoacusis and severe anxiety. The patient’s sister had depression with suicidal ideation, her father was a heavy drinker.

**Figure 1.**
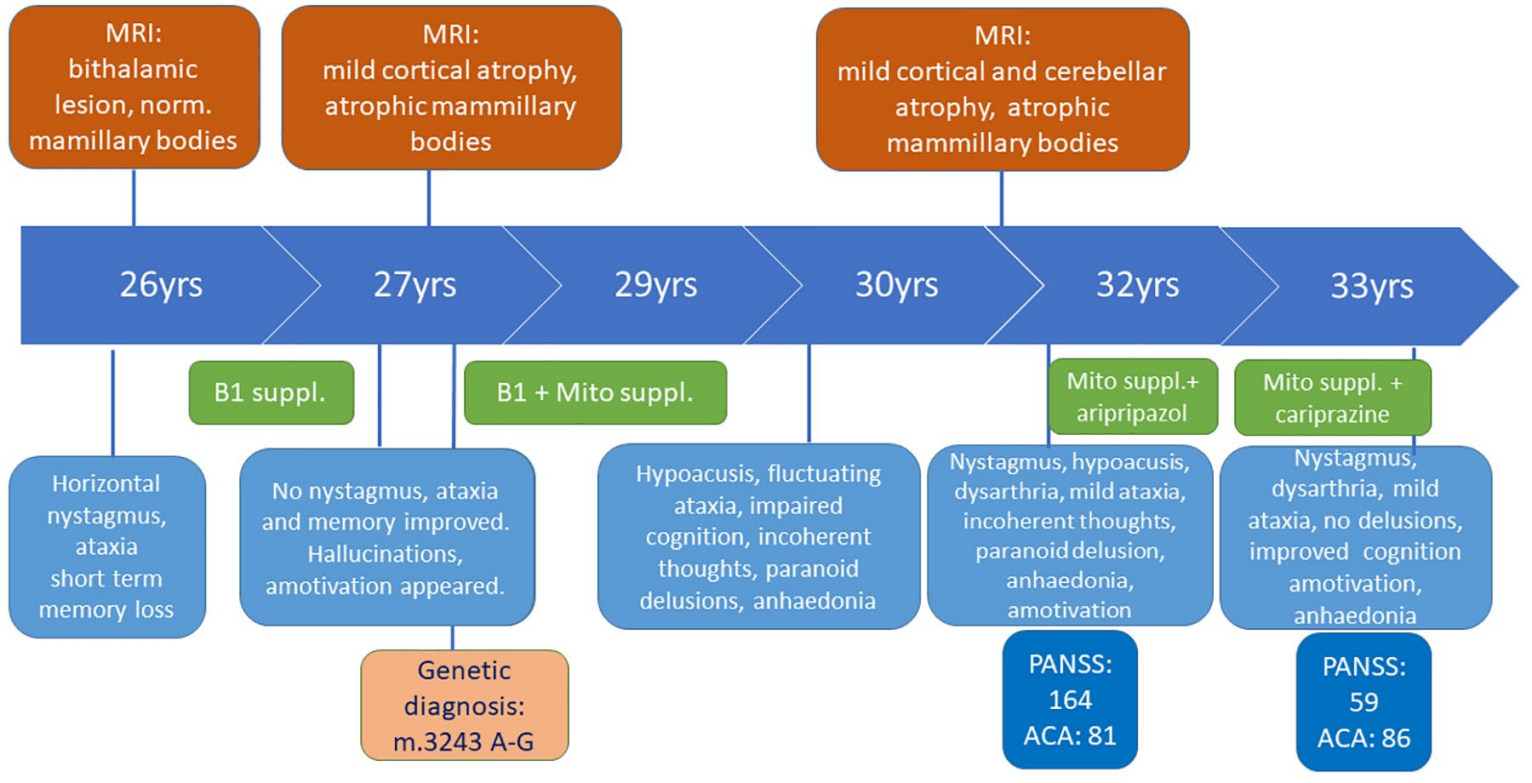
The chronology of the major clinical findings. The neurological, psychiatric status, the brain MRI findings, the major neuropsychological status and the administered medication are illustrated chronologically. ACA, Addenbrooke Cognitive Assessment; mito, mitochondrial; MRI, magnetic resonance imaging; PANSS, Positive and Negative Syndrome Scale; suppl, supplements.

**Figure 2.**
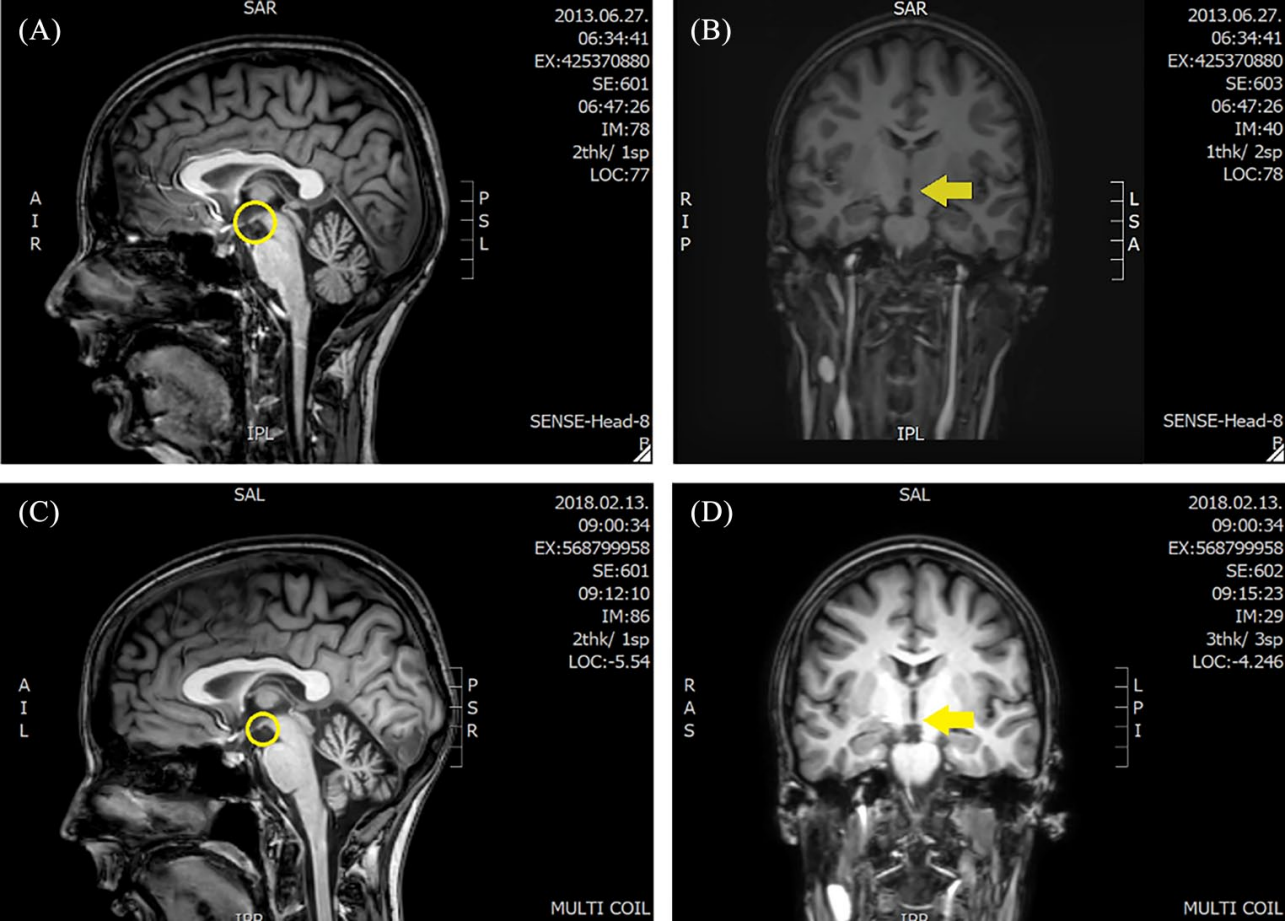
The follow up of the brain MRI. (A, B) An MRI scan was performed in 2013 on a 1.5 T MR, almost absent mamillary bodies, and widened cerebellar sulci are visible. On (C, D), which were performed in 2018 on a 3 T MR, slightly more widened cerebellar sulci and the same atrophic mammillary. MRI, magnetic resonance imaging.

Since her late teen years, she had dysmenorrhea, accompanied by diarrhoea, experienced generalized anxiety and started to use cannabis at age 17. At the age of 21, several episodes of week-long vomiting and diarrhoea occurred. No infection was discovered. Six months later, the gastrointestinal symptoms returned for a few weeks. A gluten-free diet resulted in a transitory improvement, however gluten-sensitive enteropathy was excluded, while gastroesophageal reflux disease and hepatic focal nodular hyperplasia were identified. At age 26, her prominent symptoms were ataxia, memory disturbance, fatigue and depression. High-dose parenteral vitamin B1 (200 mg/day) supplementation was started, after which the patient’s ataxia improved. The memory and learning disturbance did not change significantly, and her mood was fluctuating. The first brain magnetic resonance imaging (MRI) detected a bilateral mild thalamic lesion. Based on the clinical and MRI signs, WE diagnosis was given. A few months later at age 27, because her symptoms had progressed, she was referred to our clinic with ataxia, severe memory disturbance and confabulation associated with marked weight loss after severe vomiting episodes. Neurological examination revealed asthenic body, horizontal endpoint nystagmus, scanning speech and generalized muscle hypotrophy without paresis. Deep tendon reflexes were normal, pyramidal signs were absent and no sensory disturbance was detected. The neuropsychological tests found all aspects of memory impaired. Rey auditory verbal learning scores were 4 – 4 – 6 – 6 – 8 – 5 – 0 – 0. The Benton Visual Retention test results were also poor. Psychiatric examination revealed a severe cognitive deficit. Laboratory investigations detected anaemia, hyperglycaemia, hypoalbuminemia, hypokalaemia and elevated lactate, pancreatic and hepatic enzyme levels (Figure 1). Brain MRI showed widened cerebellar sulci and almost absent mammillary bodies on T1 sequences (Figure 2A and B). After vitamin B1 supplementation and cognitive training, the short-term memory improved, but mild auditory hallucinations and lack of initiative developed. The clinical diagnosis of WKS was made. Given the early onset of the diverse symptoms, a genetic test was performed for the most common mtDNA mutations, and an m.A3243G mutation was detected with a 42% ratio of heteroplasmy (HP) in blood. Genetic testing of her family revealed the same mutation in the mother and sister, but with a significantly lower HP ratio (15–25%) and milder phenotype. The mother had exercise intolerance, diabetes mellitus, hypoacusis and generalized anxiety, while the sister suffered from depression and personality disorder. Only the sister agreed to an MRI, which was normal. Because primary mitochondrial disease was confirmed mitochondrial supplements (two times a day: Coenzyme Q10 100 mg, L-arginine 2 gr; and once daily: Vitamin C 1 gr, B1 100 mg, B2 10 mg, B3 30 mg, D3 2000 IU, E 200 mg) – with additional dietary changes were administered. The weight loss stopped and her cognitive functions started to improve (Figure 1). At the age of 29, the patient developed hypoacusis. One year later, she had cognitive disturbances with an incoherent thinking process, signs of paranoid ideas, and impaired memory. She seemed preoccupied, her gestures were poor and she showed no emotional reactions (Figure 1).

A few years later, at the age of 32, she complained again about memory impairment, and she also reported feeling stressed. She lost her interest in routine and social activities, was unable to experience pleasure and became unemployed. Prior to this she had stopped her supportive mitochondrial medications due to financial issues. Psychiatric symptoms dominated the clinical picture: incoherent thought process, auditory hallucinations, blunted affect, lack of motivation, marked psychomotor retardation, apathy and poor self-hygiene. The Positive and Negative Syndrome Scale (PANSS) total score was 164. Further, we observed horizontal nystagmus, mild dysarthria, paresis in the lower limbs and increased deep tendon reflexes. Blind walking was slightly insecure, but feasible. The heel–shin test was normal, but the nose–finger test was inaccurate on both sides. Mixed type muscle alteration was identified by electromyography (EMG). Her lactate stress test indicated impaired aerobic metabolism. The Addenbrooke’s Cognitive Assessment (ACA) total score was 81/100 (Verbal fluency 9/14, Orientation 9/10, Concentration 8/8, Memory 22/35, Language 28/28, Space and visual skills 5/5). The Mini Mental State exam score was 29/30 (Figure 1). Aripiprazole (7.5 mg BID) therapy was started, but due to the lack of efficacy after 1 month it was switched to cariprazine (3 mg/day), which improved the psychotic and negative symptoms without any side effects. After 3 months cariprazine therapy, she started working again, was able to maintain a social life and showed gradual improvement in memory and learning skills. Half a year later, she performed 86/100 on the ACA. After a half year of cariprazine treatment, her PANSS score dropped to 59 (Figure 1). Routine laboratory results were normal and Vitamin B12 and D3 levels were decreased. The cerebellar sulci became wider, and the alteration of the mammillary bodies did not change. (Figure 2 C, D)

## Discussion

The present case describes a mA3243G mutation-associated mitochondrial encephalomyopathy, which developed to WKS and later to schizophrenia. To the best of our knowledge, this is the first case reporting this symptomatology. The patient had recurring metabolic, neurological and psychiatric symptoms. She developed psychotic symptoms multiple times in parallel to metabolic decompensations. During these psychotic episodes, negative symptoms and cognitive decline dominated the clinical picture. Herein we would like to discuss the putative genetic and molecular connections between mitochondrial dysfunction, WKS, memory loss and psychotic symptoms.

The detected mtDNA mutation is commonly associated with mitochondrial encephalopathy with lactic acidosis and stroke-like episodes (MELAS),^12^ however, it can result in a wide variety of other clinical symptoms too, such as migraine, diabetes mellitus, severe hypoacusis, ataxia, exercise intolerance and psychiatric symptoms. The presented patient was characterised by low body mass index, hypoacusis, mild dysarthria, myopathy, exercise intolerance, ataxia and cognitive impairment. No headache, stroke-like episodes or ophthalmological alterations have been observed. The family history was congruent with the disease. The threshold ratio of HP for manifesting symptoms is around 19%,^13^ however, it also depends on age. High lactate levels can be a trigger for recurrent vomiting, which can contribute to thiamine deficiency, thus MELAS may be a risk factor for WE.^14–16^

Originally, WE was associated with thiamine deficiency due to chronic alcoholism, malnutrition and malabsorption. More recently, several researches suggest a correlation between mitochondrial dysfunction, lactic acidosis as well as oxidative stress leading to selective loss of certain neurons.^16–19^ Thiamine is the cofactor of the alpha-ketoglutarate dehydrogenase (aKGDH) and transketolase enzymes of the mitochondrial citrate cycle. The lack of these enzymes alters cerebral energy utilization as aKGDH produces NADH, which fuels the OXPHOS system with electrons. In neurodegenerative diseases impairing the memory, like Alzheimer’s disease, the activity of aKGDH was found to be decreased.^20,21^ In our case, the mA3243G mutation caused metabolic disturbance, which probably led to cellular damage in regions responsible for cognitive functions, such as the mammillary bodies. Mammillary lesions are commonly seen in WKS.^22^

There is an increasing number of observations of psychiatric symptomatology in patients with mitochondrial dysfunctions.^23–25^ A report from 2007 indicates that that the prevalence of mental illnesses is 70% in adult patients with primary mitochondrial disorders.^26^ In a comprehensive case series and literature search of patients with primary mitochondrial disorders developing major psychiatric illness, m.A3243G mutation was the most common genetic diagnosis, while depression and psychosis were the most common psychiatric presentations associated with mitochondrial mutations.^23^ A larger study found that family members sharing the mtDNA with a patient diagnosed with schizophrenia had an increased risk for presenting with schizophrenia than those who did not share mtDNA.^27^ Our observations that the patient with known mitochondrial dysfunction later exhibited psychosis also support this hypothesis. Moreover, her mother and sister, carrying the same mtDNA mutation, suffered from mood and anxiety disorders too.

One possible explanation for the link between mitochondrial dysfunction and psychotic symptoms relates to the neurodevelopmental concept of schizophrenia.^28^ It is generally accepted that abnormal neural connectivity with altered glutamatergic and dopaminergic neurotransmission are responsible for the symptoms of schizophrenia.^29^ The hypofunction of glutamatergic signalling *via* NMDA receptors in schizophrenia are supported by clinical, neuropathological and genetic findings. Individuals with NMDAR autoimmune encephalitis may have psychiatric symptoms mimicking schizophrenia.^30^

Dynamic mitochondrial functioning in response to the metabolic demand is crucial for embryonic development and synapse formation. Robicsek *et al.* found that schizophrenia patient-derived stem cells associated with pervasive mitochondrial dysfunction had an impaired ability to differentiate into dopaminergic and glutamatergic neurons.^31^ One other putative explanation is that as brain cells are in a constant demand for ATP, dysfunctional mtDNA is extremely harmful for the higher-level mental functions of the brain. On the other hand, the reduced production of succinate plays a role in gamma-aminobutyric acid (GABA) metabolism.^31^ According to the GABA hypothesis of schizophrenia, decreased levels of GABA in neurotransmission cause the cognitive symptoms.^32^ Many schizophrenia EEG studies reported that patients lack gamma band activity in prefrontal areas associated with cognitive functions when compared with healthy controls.^32,33^

In the presented patient with Wernicke-like symptoms and other mitochondrial dysfunction-derived multisystemic presentations, mitochondrial supplementation, especially thiamine, improved her recurring symptoms. However, neural and muscle conditions slowly progressed over time. For the manifesting schizophrenia symptoms, the therapeutic choice was challenging considering the prominent negative symptoms and metabolic background of the patient. Cariprazine significantly mitigated both positive and negative symptoms, improved everyday functioning and had no metabolic or other side effects.

## Conclusion

In the presented case, we assume that patients with mtDNA disorders are more prone to develop WKS due to metabolic alterations. In these cases, besides parenteral thiamine supplementation, mitochondrial supplements can be useful in treating WKS and to prevent exacerbations. In patients having prominent negative symptoms associated with mitochondrial dysfunction, cariprazine may be an effective and safe symptom-based, personalized, antipsychotic treatment option. Cariprazine showed no side effects and was able to control schizophrenia symptoms as well as cognition and functioning.
